# Supplementary material for: A less cloudy picture of the inter-model spread in future global warming projections
Source: Nat Commun. 2020 Sep 8;11:4472. doi: 10.1038/s41467-020-18227-9 (PMC7479110; doi:10.1038/s41467-020-18227-9)
Supplement: Supplementary file 1 — Supplementary Information for A Less Cloudy Picture of the Inter-Model Spread in Future Global Warming Projections [file 41467_2020_18227_MOESM1_ESM.pdf]

Supplementary Information for

A Less Cloudy Picture of the Inter-Model Spread in Future Global Warming Projections

Xiaoming Hu[1,2], Hanjie Fan[1], Ming Cai[3*], Sergio A. Sejas[4], Patrick Taylor[5], Song Yang[1,2]

[1]School of Atmospheric Sciences, Sun Yat-sen University, Guangzhou, Guangdong 510275, China.

[2]Southern Marine Science and Engineering Guangdong Laboratory (Zhuhai), Zhuhai, Guangdong 519082, China

[3]Department of Earth, Ocean and Atmospheric Sciences, Florida State University, Tallahassee, FL 32304, USA.

[4]Science Systems and Applications, Inc, Hampton, VA 23666, USA.

[5]Climate Science Branch, NASA Langley Research Center, Hampton, VA 23681, USA.

*To whom correspondence should be addressed. E-mail: mcai@fsu.edu

Supplementary Table 1: A list of CMIP5 models analyzed in this study in the order of their projected global mean surface temperature change (<ΔT>) from the smallest to the largest. <ΔT> is defined as the global mean difference between the RCP8.5 (the mean of 2051-2100) and historical run (the mean of 1951-2000) experiments. <ΔT*> is the global mean warming projection spread (WPS) or the departure of <ΔT> of individual models from the multi-model ensemble mean, equaling 3.20 K.

	**Model acronym**	**Institution**	<ΔT>	<ΔT*>
**1**	GISS-E2-R	NASA-Goddard Institute for Space Studies	2.22	-0.93
**2**	GFDL-ESM2M	NOAA-Geophysical Fluid Dynamics Laboratory	2.32	-0.83
**3**	GFDL-ESM2G	NOAA-Geophysical Fluid Dynamics Laboratory	2.46	-0.70
**4**	FGOALS-g2	Institute of Atmospheric Physics, Chinese Academy of Sciences, and Tsinghua University	2.56	-0.59
**5**	GISS-E2-H	NASA-Goddard Institute for Space Studies	2.59	-0.57
**6**	MRI-CGCM3	Meteorological Research Institute	2.75	-0.41
**7**	NorESM1-M	Norwegian Climate Centre	2.77	-0.38
**8**	MIROC5	The University of Tokyo, National Institute for Environmental Studies, and Japan Agency for Marine-Earth Science and Technology	2.89	-0.26
**9**	NorESM1-ME	Norwegian Climate Centre	2.93	-0.22
**10**	IPSL-CM5B-LR	Institute Pierre-Simon Laplace	2.95	-0.21
**11**	MPI-ESM-MR	Max Planck Institute for Meteorology	3.03	-0.13
**12**	MPI-ESM-LR	Max Planck Institute for Meteorology	3.04	-0.11
**13**	CMCC-CESM	Centro Euro-Mediterraneo per I Cambiamenti Climatici	3.16	0.00
**14**	bcc-csm1-1	Beijing Climate Center	3.17	0.02
**15**	CESM1-BGC	NSF/DOE, National Center for Atmospheric Research	3.19	0.03
**16**	CCSM4	National Center for Atmospheric Research	3.26	0.10
**17**	CSIRO-Mk3.6.0	CSIRO in collaboration with Queensland Climate Change Centre of Excellence	3.28	0.12
**18**	CMCC-CM	Centro Euro-Mediterraneo per I Cambiamenti Climatici	3.45	0.30
**19**	CESM1-CAM5	NSF/DOE, National Center for Atmospheric Research	3.56	0.40
**20**	CMCC-CMS	Centro Euro-Mediterraneo per I Cambiamenti Climatici	3.57	0.41
**21**	BNU-ESM	College of Global Change and Earth System Science, Beijing Normal University	3.82	0.67
**22**	IPSL-CM5A-MR	Institute Pierre-Simon Laplace	3.89	0.74
**23**	IPSL-CM5A-LR	Institute Pierre-Simon Laplace	3.96	0.81
**24**	GFDL-CM3	NOAA-Geophysical Fluid Dynamics Laboratory	4.00	0.85
**25**	CanESM2	Canadian Centre for Climate Modelling and Analysis	4.03	0.88

Supplementary Table 2: A list of CMIP5 model simulations that are not analyzed in this study for the reason specified on the right column.

	Model acronym	Institution	Reason why not used
**1**	ACCESS1-0	CSIRO and Bureau of Meteorology	no enough information to interpolate cloud data
**2**	ACCESS1-3		
**3**	CNRM-CM5	Centre National de Recherches M´et´eorologiques	no multi-level cloud data
**4**	HadGEM2-CC	Met Office Hadley Centre	no enough information to interpolate cloud data
**5**	HadGEM2-ES		
**6**	inmcm4	Institute for Numerical Mathematics	missing cloud data
**7**	FGOALS-s2	Institute of Atmospheric Physics, Chinese Academy of Sciences	Pronounced inconsistency in changes of cloud fields with all other models

Supplementary Table 3: A list of model variables/parameters used in this study.

	Short name	Long name	Vertical resolution*	Units
**1**	cl	Cloud Area Fraction	P	1
**2**	cli	Mass Fraction of Cloud Ice	P	1
**3**	clw	Mass Fraction of Cloud Liquid Water	P	1
**4**	hus	Specific Humidity	P	1
**5**	ta	Air Temperature	P	K
**6**	tro3	Mole Fraction of O3	P	1
**7**	ps	Surface Air Pressure	S	Pa
**8**	huss	Near-Surface Specific Humidity	S	1
**9**	ts	Surface Temperature	S	K
**10**	rsds	Surface Downwelling Shortwave Radiation	S	W/m^2
**11**	rsus	Surface Upwelling Shortwave Radiation	S	W/m^2
**12**	rsdt	TOA Incident Shortwave Radiation	S	W/m^2
**13**	hfls	Surface Upward Latent Heat Flux	S	W/m^2
**14**	hfss	Surface Upward Sensible Heat Flux	S	W/m^2

* Here “P” denotes standard pressure level data (1000 hPa, 925 hPa, 850 hPa, 700hPa, 600 hPa, 500 hPa, 400 hPa, 300 hPa, 250 hPa, 200 hPa, 150 hPa, 100 hPa, 70 hPa, 50 hPa, 30 hPa, 20 hPa, 10 hPa) and “S” denotes the single level data at the surface.

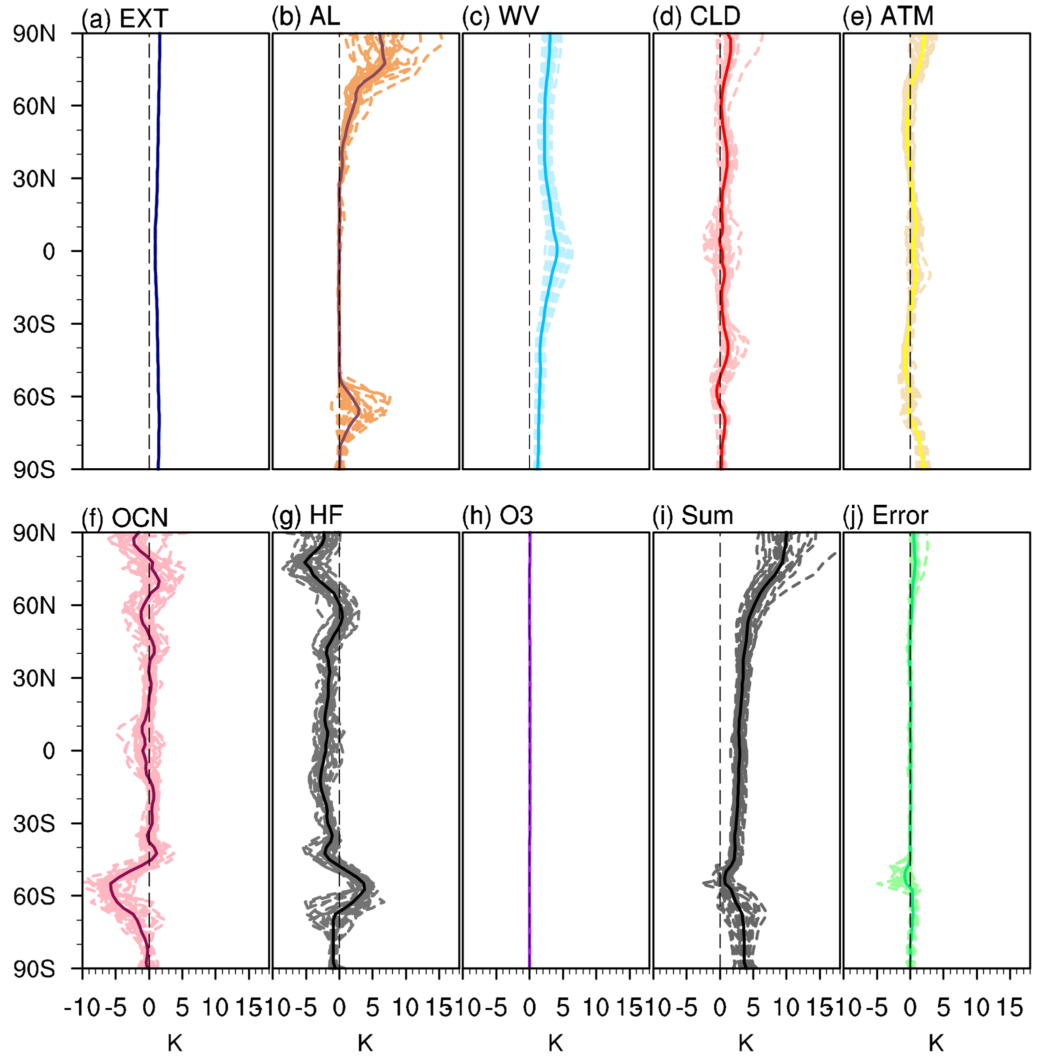

Supplementary Figure 1. **Partial Surface Temperature Changes**. Zonal means of CFRAM-derived partial surface temperature changes given by all 25 models. Panels (a)–(h) are CFRAM-decomposed partial surface temperature changes due to changes in, respectively, the external forcing (EXT), surface albedo (AL), water vapor (WV), clouds (CLD), atmospheric dynamics (ATM), ocean dynamics and heat storage (OCN), surface heat fluxes (HF), and ozone (O3). Panel (i) is the sum of (a)–(h) and (j) is the difference between Fig. 1b and the panel (i), corresponding to the error of the CFRAM analysis. The solid line in each panel is the MME mean. Note that what is shown here is the same as that shown in Figs. 1d–1m but without removing their multi-model ensemble means.

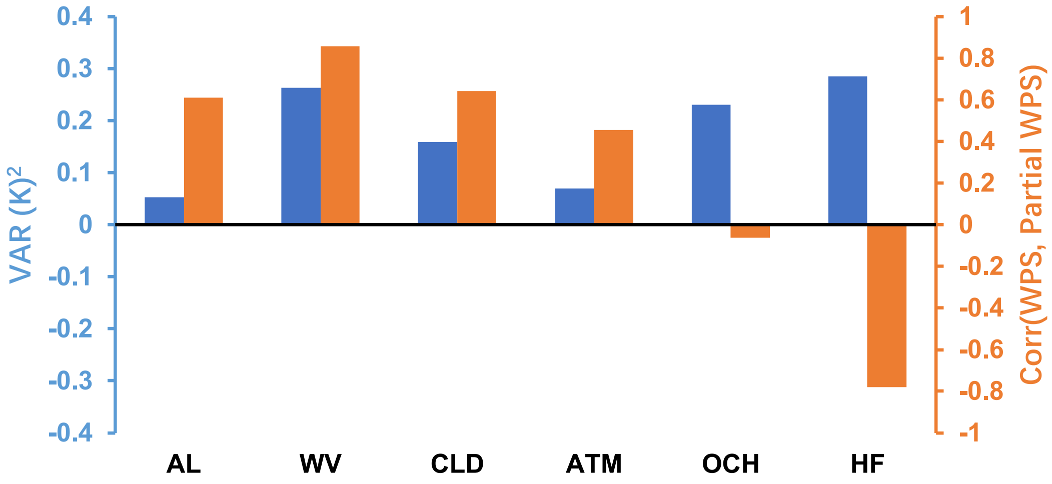

Supplementary Figure 2. Process Correlations with the GWS. Variance of zonal mean inter-model spreads of partial surface temperature changes due to individual feedback processes (blue, the ordinate on the left) and the correlations of their global means (orange, the ordinate on the right) with the global mean warming spread (GWS). The labels on the bottom of each panel stand surface albedo (AL), water vapor (WV), clouds (CLD), atmospheric dynamics (ATM), ocean dynamics/storage (OCN), and surface fluxes (HF) feedbacks.

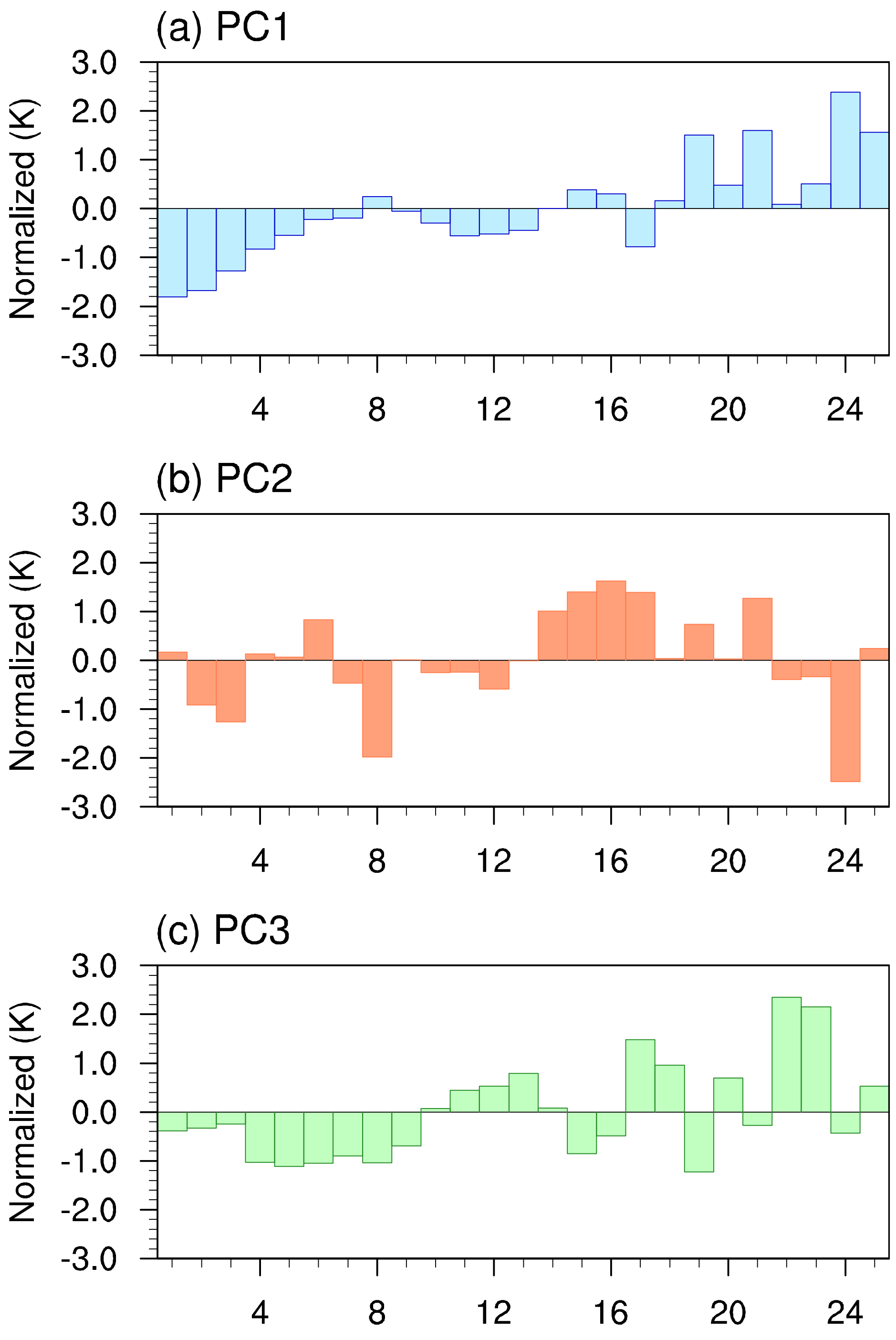

Supplementary Figure 3. Principal Components of the Dominant EOF Modes. The ordinate corresponds to the normalized principal components (dimensionless) of the (a) EOF1, (b) EOF2, and (c) EOF3 of the zonal mean inter-model warming projection spread. The abscissa corresponds to the model number. The standard deviation of the normalized principal component by definition is equal to one.

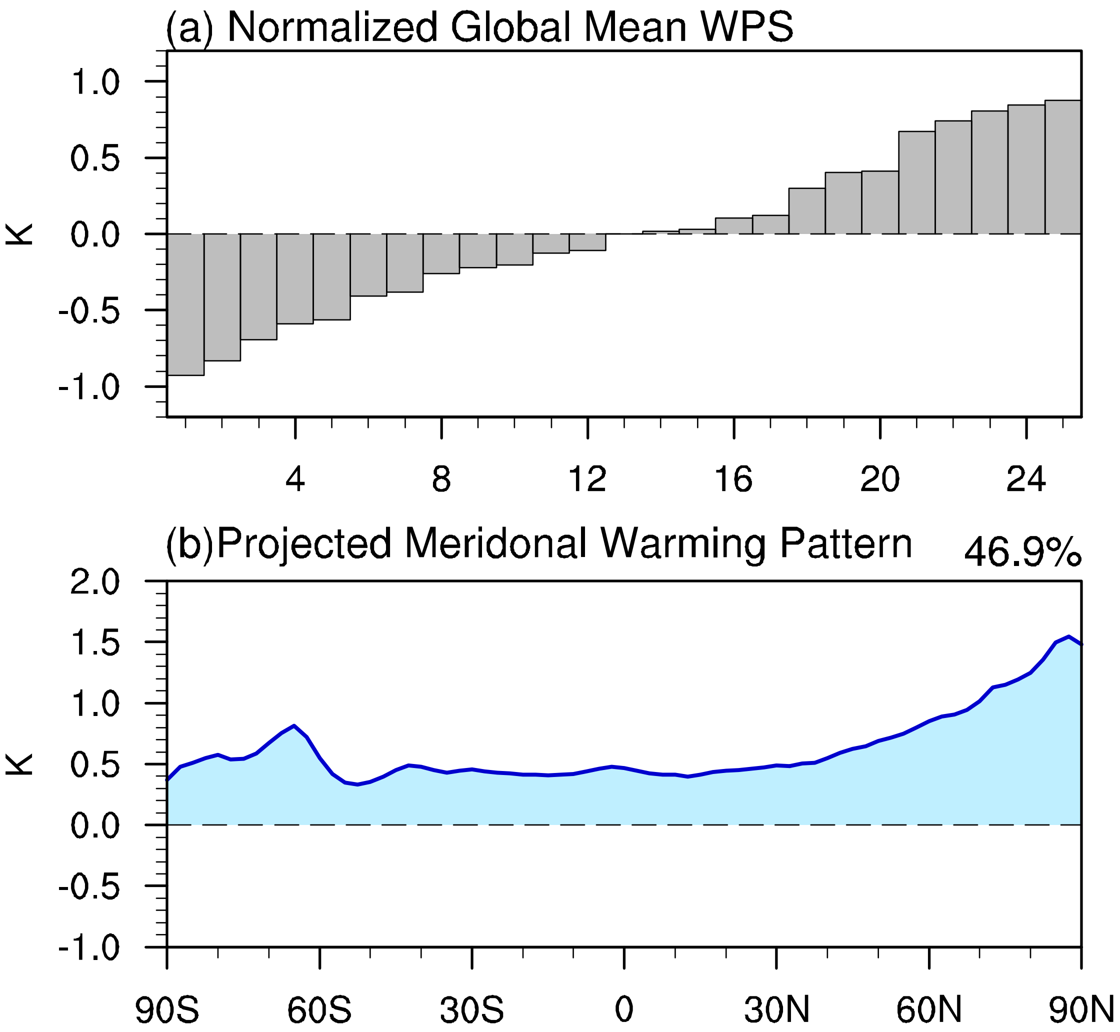

Supplementary Figure 4. Regressed Zonal Mean Warming Pattern against the GWS. (a) The normalized global mean departure of the surface temperature change from the multi-model ensemble (MME) mean (ordinate; dimensionless) for individual models (abscissa) and (b) the zonal mean warming pattern (ordinate; K) obtained by regressing the curves shown in Fig. 1c against the series shown in panel (a) above. The normalization factor used in (a) is the standard deviation of the global means of the inter-model warming projection spread. The percentage number in (b) corresponds to the percentage of the total variance in the zonal mean inter-model WPS explained by the spatial pattern in (b).

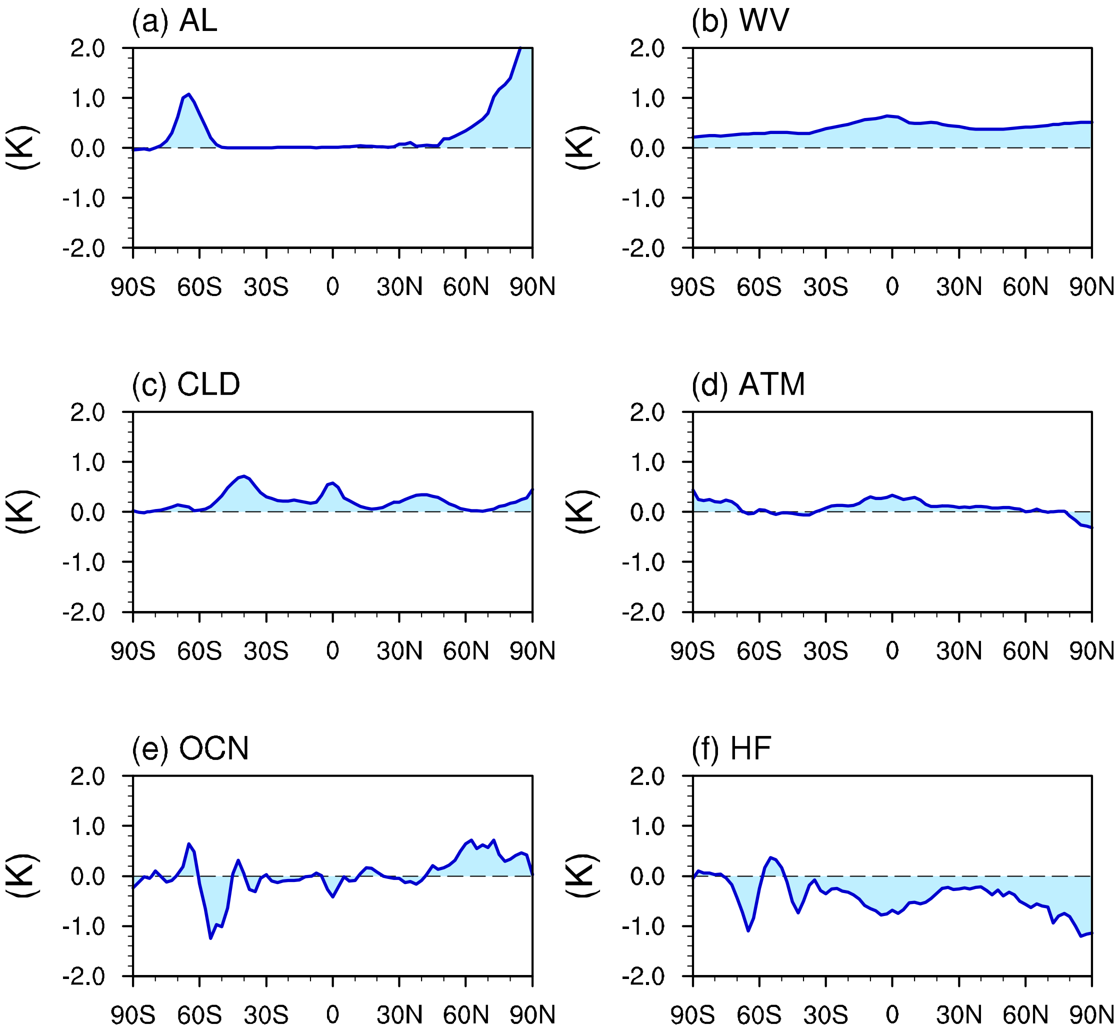

Supplementary Figure 5. Regressed Zonal Mean Inter-Model Spreads of Partial Temperature Changes against the GWS. The meridional patterns obtained by regressing the inter-model spreads of zonal mean partial surface temperature changes (ordinate, K), associated with (a) surface albedo (AL) feedback, (b) water vapor (WV) feedback, (c) clouds (CLD) feedback, (d) atmospheric dynamics (ATM) feedback, (e) ocean dynamics/storage (OCN) feedback, and (f) surface fluxes (HF) feedback, against the normalized global mean warming projection spread (i.e., the series in (a) of **Supplementary** Figure 4).

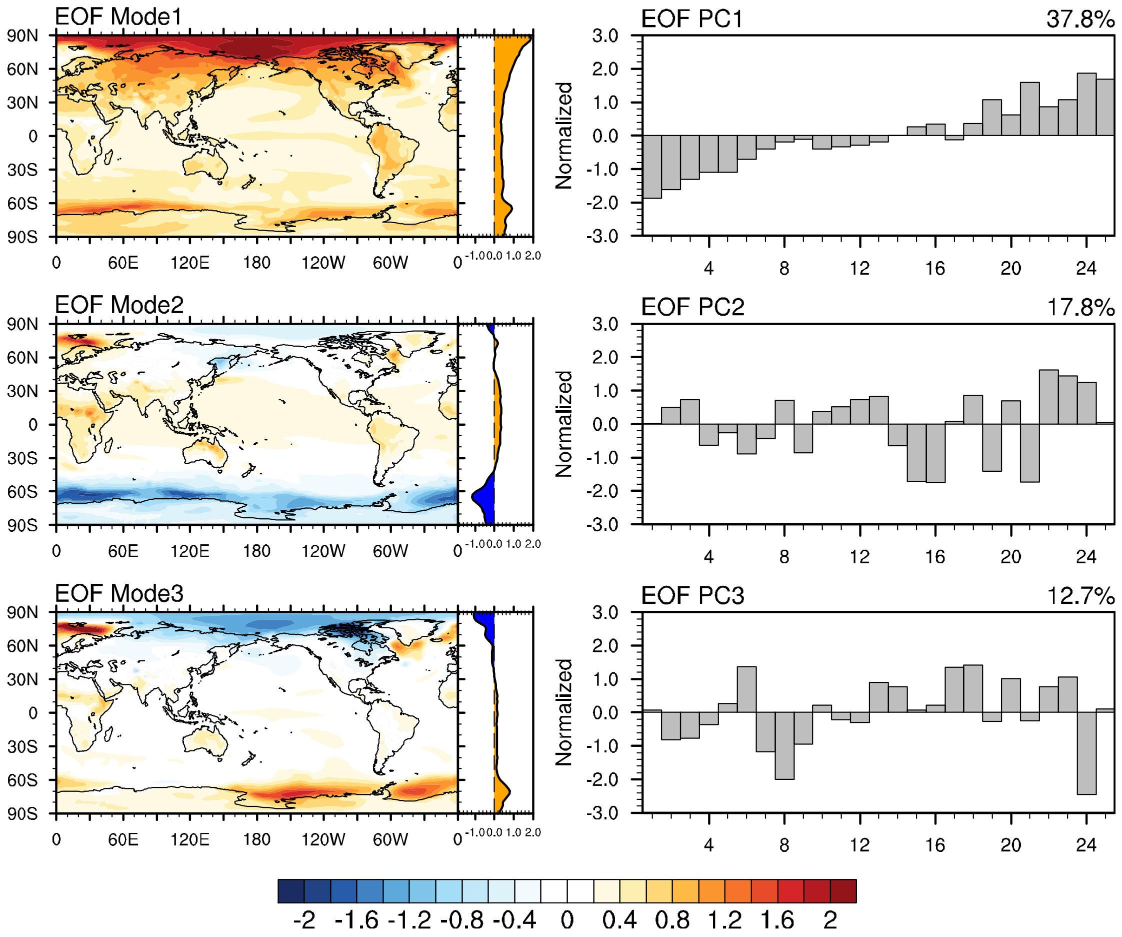

Supplementary Figure 6. 2-D EOF Analysis of the 2-D WPS. Spatial patterns of the first three EOF modes (K) that explain the majority of the variance in the inter-model warming projection spread (left column) and their principal components (right column). The standard deviation of the (dimensionless) principal components is equal to one. The right portion of the left column corresponds to the zonal means of the maps on the left.

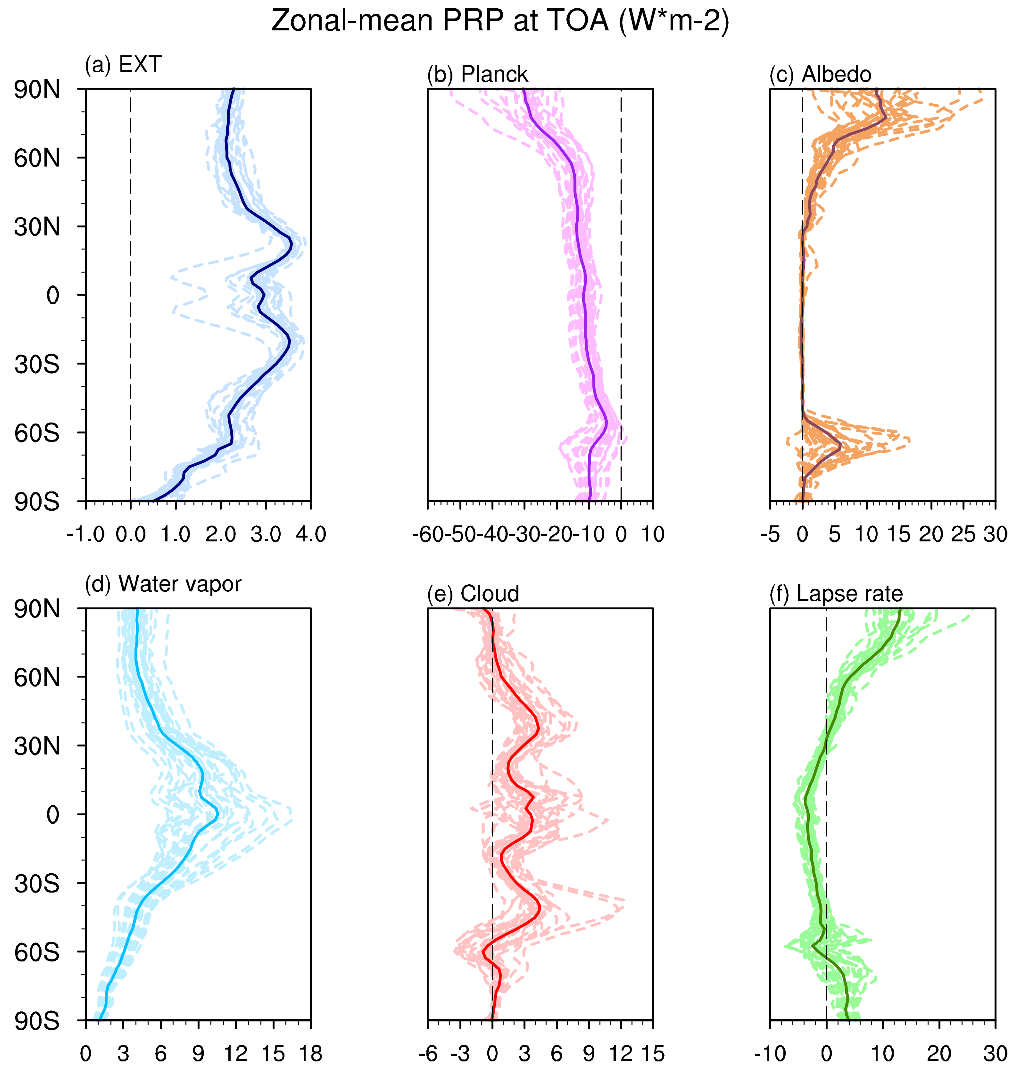

Supplementary Figure 7. Partial Radiative Energy Flux Perturbations at the TOA. Zonal means of partial radiative energy flux perturbations (PRP, W/m^2) given by all 25 models. Panels (a)–(f) are PRP associated with, respectively, the external forcing (EXT), Planck feedback (PL), surface albedo feedback (AL), water vapor feedback (WV), cloud feedback (CLD), and lapse rate feedback (LR). The solid line in each panel is the MME mean.

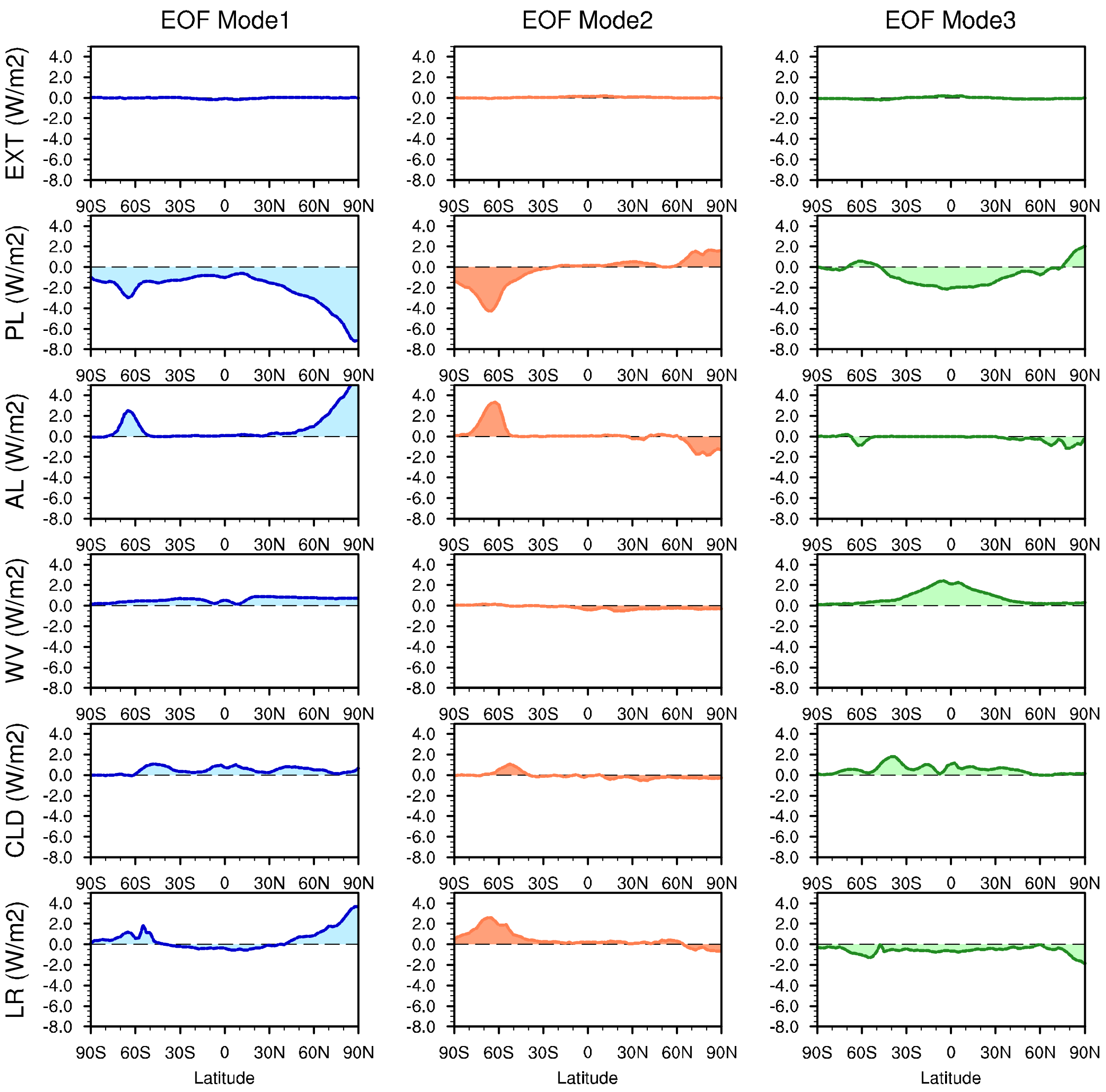

Supplementary Figure 8. Regressed Patterns of Inter-model Spreads in PRP at TOA. Shown in the three columns are the regressed patterns against the principal components of EOF1 (the left column), EOF2 (the middle column), and EOF3 (the right column). Shown from the top to the bottom rows are, respectively, the regress patterns of the zonal means of partial radiative energy flux perturbations (W/m^2) due to the external forcing (EXT), Planck feedback (PL), surface albedo feedback (AL), water vapor feedback (WV), cloud feedback (CLD), and lapse rate feedback (LR).

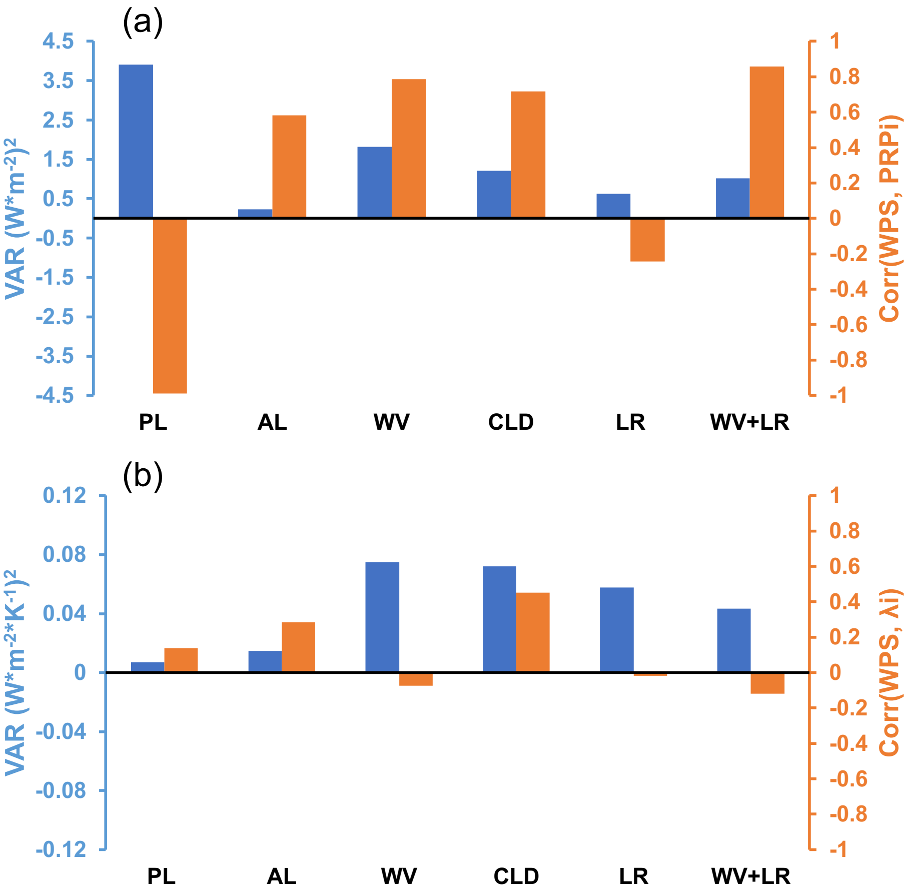

Supplementary Figure 9. Variance and Correlation of the GWS with spreads of individual climate feedbacks at the TOA. Variance is shown as blue bars with ordinate on the left and the correlation as orange bars with the ordinate on the right. Shown in Panel (a) are the results obtained from the inter-model spread of the global mean partial radiative flux perturbations at the TOA due to individual processes and (b) the results obtained from the inter-model spread of climate feedback parameters. The labels on the bottom of each panel stand for Planck feedback (PL), surface albedo feedback (AL), water vapor feedback (WV), cloud feedback (CLD), lapse rate feedback (LR), and the sum of water vapor and lapse rate feedbacks (WV+LR).
